# Effectiveness of a day program for patients with eating disorders

**DOI:** 10.1186/2050-2974-1-S1-O3

**Published:** 2013-11-14

**Authors:** Susan Hart, Sarah Horsfield, Angela Nickerson, Janice Russell, Chris Thornton, Sarah Maguire

**Affiliations:** 1 Department of Psychiatry, Royal Prince Alfred Eating Disorder Day Program, Australia; 2 School of Psychology, University of New South Wales, Australia; 3 The Redleaf Practice, Australia; 4 The Centre for Eating and Dieting Disorders, The University of Sydney, Australia

## 

Derwent House is a government funded pilot treatment day program within an established eating disorder service that commenced operation in 2010. It is run by a multidisciplinary team of clinical psychologists, dietitians and an occupational therapist. The program is based on a cognitive behaviour therapy framework with patients attending four days per week. Admission lengths range from four to twelve weeks depending on their individual treatment plan. Fifty patients completed outcome measures at admission, discharge, six and twelve month follow-up. Measures collected included the Eating Disorder Examination Questionnaire; the Eating Attitudes Test; the Depression, Anxiety and Stress Scale; the Eating Disorder Quality of Life instrument; the Intuitive Eating Scale; the Rosenberg Self-Esteem Scale; and the Anorexia/Bulimia Stages of Change Questionnaires. The data has been analysed using hierarchical linear modelling. Findings suggested that this intervention was effective in reducing eating disorder symptoms. Results on the short and long-term treatment effectiveness of the day program will be presented.

This abstract was presented in the **Adult Treatment and Services** stream of the 2013 ANZAED Conference.

